# Effects of Childhood Adversity and Its Interaction with the MAOA, BDNF, and COMT Polymorphisms on Subclinical Attention Deficit/Hyperactivity Symptoms in Generally Healthy Youth

**DOI:** 10.3390/children7090122

**Published:** 2020-09-03

**Authors:** Meng-Che Tsai, Kai-Jyun Jhang, Chih-Ting Lee, Yu-Fang Lin, Carol Strong, Yi-Ching Lin, Yi-Ping Hsieh, Chung-Ying Lin

**Affiliations:** 1 Department of Pediatrics, National Cheng Kung University Hospital, College of Medicine, National Cheng Kung University, Tainan 70101, Taiwan; fabian800128@icloud.com (K.-J.J.); mandylin79@hotmail.com (Y.-F.L.); 2 Department of Family Medicine, National Cheng Kung University Hospital, College of Medicine, National Cheng Kung University, Tainan 70101, Taiwan; christina.unicorn@gmail.com; 3 Department of Public Health, National Cheng Kung University Hospital, College of Medicine, National Cheng Kung University, Tainan 70101, Taiwan; carol.chiajung@gmail.com; 4 Department of Early Childhood and Family Education, College of Education, National Taipei University of Education, Taipei 10671, Taiwan; ylin11@mail.ntue.edu.tw; 5 Department of Social Work, College of Nursing and Professional Disciplines, University of North Dakota, Grand Forks, ND 58202, USA; yiping66@gmail.com; 6 Department of Rehabilitation Sciences, Hong Kong Polytechnic University, Hung Hom, Hong Kong; cylin36933@gmail.com

**Keywords:** childhood adversity, attention deficit, hyperactivity, impulsiveness, gene-environment interaction

## Abstract

We aimed to investigate the effects of childhood adversity and its interaction with the polymorphisms in the monoamine oxidase A (MAOA), brain-derived neurotrophic factor (BDNF), and catechol-O-methyltransferase (COMT) genes on attention and hyperactivity disorder (ADHD) symptoms in a community sample of generally healthy youth. Participants (N = 432) completed questionnaires assessing ADHD symptoms (i.e., inattention, hyperactivity, and impulsiveness) and adverse childhood experiences, such as adverse environments (AEs) and childhood maltreatment (CM). Salivary genomic DNA was used to test polymorphisms in MAOA, BDNF, and COMT genes. A gene score (GS) was created based on the number of risk allele in the studied genes. Multiple linear regressions were used to examine the genetic and environmental effects on ADHD symptoms. The univariate analysis indicated that CM was significantly associated with inattention (β = 0.48 [95% confidence interval 0.16–0.79]), hyperactivity (0.25 [0.06–0.45]), and impulsiveness (1.16 [0.26–2.05]), while the GS was associated with hyperactivity (0.22 [0.11–0.33]) and impulsiveness (0.56 [0.06–1.05]). Only the GS remained significantly associated with hyperactivity (0.25 [0.12–0.37]) and impulsiveness (0.79 [0.20–1.38]) when the gene-environment interaction term was added in the model. No effects were found for AE and the gene-environment interaction term. In conclusion, CM was associated with ADHD symptoms in emerging adulthood. Genetic factors may also play a significant role in the association with these outcomes.

## 1. Introduction

Attention deficit hyperactivity disorder (ADHD) is a common neurodevelopmental disorder that has heterogeneity in its etiology [1]. Prior research has demonstrated etiological heterogeneity in terms of multiple genetic and environmental risk factors that might interact with each other and thus result in the diverse cognitive and behavioral trajectories of the disorder through a complex developmental neural mechanism [2]. ADHD has long been thought of as having an early onset specifically in childhood and continuing into adolescence in some affected individuals, and it is thus required that symptoms must be present before the age of 12 years, in order to fulfill criteria for a diagnosis of ADHD. From the perspective of the ontogenic process, a genetically shared impulsive trait may be expressed as ADHD very early in life and progress to a wide spectrum of externalizing behavioral problems across development [3]. However, accumulative evidence raises the possibility that ADHD symptomatology emerging in young adulthood may be distinct from a diagnosis of ADHD itself and represent a distinctive entity, though this distinction is not currently clear, nor is the difference between the short-and long-term impact of having an ADHD diagnosis versus symptoms that requires further research and clinical attention [4]. Compared to the majority of research attention paid to child cohorts, studies dedicated to the emerging ADHD symptoms in young adults are relatively scarce.

Earlier studies have found that psychosocial and familial environmental adversity is likely to increase the risk of ADHD symptoms [5,6] as well as impulsive behaviors [7] in children and adolescents. For instance, Biederman et al. [6] found a dose-dependent manner in the association between the levels of environmental adversity (including family conflict, social class, family size, maternal psychopathology, and paternal criminal) and the risk for ADHD. In addition, individuals who have been exposed to various types of maltreatment in early childhood have been shown to be at greater risk of ADHD symptoms, and increased exposure to maltreatment has been shown to predict symptom persistence [8]. Large-scale longitudinal studies also have demonstrated that childhood maltreatment may be associated with ADHD symptoms in young adulthood [9,10]. While the timing of childhood maltreatment is usually defined by its occurrence before age 18 in most adult research, Capusan et al.’s study [9] further highlighted that earlier childhood maltreatment with occurrence before age 7 was independently associated with ADHD symptoms in adulthood even after adjusting for re-traumatization in middle childhood and adolescence. However, the mechanistic pathway linking the childhood adversity and subsequent ADHD symptoms remains unclear. One of the explanations is that childhood trauma may alter the central nervous network, particularly in the frontal region that regulates executive functions related to inhibitory behavioral control [11]. This link due to the central nervous system may not sufficiently explain why some individuals can overcome stress related to adverse experiences while some cannot and thus develop ADHD symptoms [12]. Whether individual genetic compositions confer risk and/or protective factors for ADHD symptoms and how childhood adversity interacts with the genetic predispositions continue to intrigue developmental researchers [13]. Another plausible explanation may be gene-environment interaction, where it has been posited that an early home environment is a factor that may modify the expression of genes potentially implicated in ADHD pathogenesis, and therefore, childhood adversity may increase the severity of extant ADHD symptoms [14,15]. 

ADHD has been genetically linked to neurological dysfunctions, including involvement in the dopaminergic, adrenergic, serotoninergic, and cholinergic pathways [16]. For instance, genetic polymorphisms in the monoamine oxidase A (MAOA) [17], brain-derived neurotrophic factor (BDNF) [18], and catechol-O-methyltransferase (COMT) [19] genes have been widely reported in association with ADHD symptoms. However, conflicting results exist among different variant locations and ADHD subtypes [20,21]. Since genetic and environmental factors are not working completely independently in terms of the occurrence of ADHD [22], etiological explanations may rely on the interactive effects of these two crucial factors. A few studies have found that some genetic variants in the dopaminergic or serotoninergic pathway may interact with adverse childhood experiences (ACEs) via moderating the relationship, with subsequent ADHD occurrences [23,24]. In addition, recent genetic association analyses have shifted the focus onto the accumulative effects across multiple single nucleotide polymorphisms (SNPs) [25]. New evidence may evolve from the combination effects of the target genetic markers in genetic association studies of ADHD occurrence [26].

ADHD symptoms are not uncommon but are often overlooked among those entering adulthood [27]. Higher levels of self-reported ADHD symptoms have been closely linked to adverse health impacts, such as suicidal ideation, emotional disturbance, and additive behaviors in the young adult population [28]. Current knowledge about emerging adult ADHD symptoms is patchy, and the determinants also remain largely unclear. To fill this gap, this paper was aimed toward empirically testing the gene-environment interaction model of ADHD occurrence. Specifically, we examined the effects of childhood adversity and its interaction with polymorphisms in the MAOA, BDNF, and COMT genes on subsequent ADHD symptoms in emerging adulthood.

## 2. Materials and Methods

### Participants

A convenience sample of young people aged 16–22 years were voluntarily recruited from the campus or community via research announcements. Peeople were excluded if they had major physical (e.g., congenital syndromes, mental retardation and epilepsy) or psychiatric (e.g., bipolar disorder, major depression and schizophrenia) diagnoses. Individuals with prior clinically confirmed diagnosis of ADHD were also excluded, as their symptoms may have onset in childhood and persist into adolescence or young adulthood. As such, our sample can be more representative of generally healthy young people. Participants and their guardians (if participants’ age was under age 20) were informed and gave consent as they completed the questionnaire. Those who were unable to provide informed consents were also excluded from the study. The entire procedure was approved by the institutional review board of our institution (B-BR-104086).

## 3. Measurements

### 3.1. Attention Deficit and Hyperactivity Symptomatology

ADHD symptoms were assessed using the six-item Adult ADHD Self-Report Scale (ASRS-6). The Chinese version of ASRS-6 has been validated in a young Taiwanese population [29]. The scale comprised 4 items for attention deficit (i.e., “having trouble in wrapping up the final details”, “having difficulty in getting things in order”, “have problems remembering appointments or obligations”, and “avoiding or delaying getting a task started”) and 2 items for hyperactivity (i.e., “fidgeting or squirming with your hands or feet when you have to sit down for a long time”, and “feel overly active and compelled to do things”). The items were scored on a 4-point Likert’s scale. In the present study, attention deficit (range: 4–16) and hyperactivity (range: 2–8) were analyzed separately using a sum score of items loaded in the subscale.

### 3.2. Impulsiveness

A short Chinese version of impulsiveness scale developed from the Baratt Impulsive Scale to evaluate the participants’ impulsive characteristics has been validated in this local context [30]. We adopted the subscale of motor impulsiveness (range: 15–60), which comprises 15 items rated on a 4-point Likert’s scale, to complement the capture of entire core adult ADHD symptoms, given ASRS-6 only able to assess hyperactive symptoms. The Cronbach α was 0.79 in our study cohort. 

### 3.3. Adverse Childhood Experience

We adopted a modified version of the Adverse Childhood Experience International Questionnaire (ACE-IQ) to assess the ACE type and timing of the participants [31]. The tool included a total of 14 questions on different ACEs, ranging from adverse environments (AEs) and childhood maltreatments (CMs) [32]. The eight items related to AE were retrieved from those on family environment in ACE-IQ, including parental marriage status, forced displacement, witnessing domestic violence and alcoholism mental illness, illicit drug use, incarceration, and death among household members, while the other six items related to CM were malicious injury, sexual harassment, neglect, and physical, verbal, and sexual abuse [32]. Aligned with prior research where CMs were particularly implicated in their association with adult ADHD symptoms [9,10], answers were dichotomized for each ACE type, and a sum score was calculated for AE and CM. In the questionnaire, participants were asked to indicate whether CMs ever occurred before the entry of elementary school (around age 6 up to 7), a memorable transition point in childhood life. As such, it allowed us to further examine the effect of earlier exposure to maltreatment as a sensitive analysis in line with prior research [9]. We identified and calculated a separate sum score for CMs occurring before the age of 6 years (CM6). Given the 12-month spread that exists in the age of entry to elementary school, CM6 operationalized in our analysis was presumed to capture early maltreatments that were comparable to those defined by the occurrence before age 7 in Capusan et al.’s study [9]. We analyzed these environmental factors, i.e., AE, CM, and CM6, separately.

### 3.4. MAOA, BDNF, and COMT Polymorphisms

We selected representative SNPs in the MAOA (rs1137070), BDNF (rs6265), and COMT (rs4680) genes. Salivary genomic DNA was extracted according to the standardized protocol (Geneius™ Micro gDNA Extraction Kit). Genotypes at the respective SNPs were determined for each sample using real time PCR that followed the standardized Taqman technology protocol (Applied Biosystems). Individuals with missing values in more than one SNP were eliminated from the analysis, as the combination effects of the three SNPs needed to be tested [20]. For analytical purposes, we tested three genetic inheritance models, i.e., dominant, recessive, and additive models. The genotypes were binary in the dominant and recessive models, while the number of minor alleles was treated as a dosage scale in the additive model. 

## 4. Statistical Analysis 

First, we tested individual associations between the selected SNPs and ADHD symptoms in three different inheritance models using linear regression analyses. In additive models, a beta coefficient (β) with 95% confidence interval (CI) was obtained for the minor allele vs the major allele. Further, based on an additive inheritance model, we created a gene score (GS, ranging from 0 to 6) by adding up the number of risk alleles, which was defined by the allele that was positively associated with the outcome variables. To test the genetic, environmental (i.e., ACEs), and gene-environment interaction effects on the outcome variables, we built three different linear regression models. In Model 1, we tested the univariate effect of GS or ACEs on the outcome variables. Model 2 included GS and ACEs at the same time, while Model 3 further included the interaction term between the GS and ACEs. Missing data were imputed. As patterns of data did not differ before and after imputation, we only presented the results after imputing the data. All analyses were adjusted for age and gender, and a gender-stratified analysis was also conducted. Statistical analyses were performed using R Studio 1.2.1335. 

## 5. Results

A total of 492 participants were recruited and, after eliminating those with missing values in more than one SNP, only 432 with a mean age of 20 (±1.41) years and 51% being males were considered valid for analysis (Table 1). The mean score (±standard deviation) was 8.3 (±2.4) for inattention, 4.0 (±1.5) for hyperactivity, and 31.4 (±6.7) for impulsiveness. The average counts were 0.66 (±0.98) for AE, 0.50 (±0.70) for CM, and 0.27 (±0.53) for CM6. After adjustment for gender and age (Table 2), the C allele in the MAOA polymorphism was shown to be negatively associated with impulsiveness in a recessive model (β = −1.36, [95% CI −2.7, −0.01]). The C allele in the BDNF polymorphism was consistently associated with hyperactivity in the dominant (β = 0.38, [0.06, 0.71]), recessive (β = 0.35, [0.01, 0.7]), and additive models (β = 0.29, [0.08, 0.49]). It was also associated with impulsiveness in the additive model (β = 0.97, [0.04, 1.91]). As for the A allele in the COMT polymorphism, a negative association was found in the link to hyperactivity in the dominant (β = −0.36, [−0.64, −0.07]) and additive models (β = −0.24, [−0.45, −0.03]).

We further applied GS to test the genetic, environmental, and gene-environment interaction effects (Table 3). In the univariate analysis (Model 1), CM was significantly associated with inattention (β = 0.48, [0.16, 0.79]), hyperactivity (β = 0.25, [0.06, 0.45]), and impulsiveness (β = 1.16, [0.26, 2.05]), while the GS was associated with hyperactivity (β = 0.21, [0.11, 0.32]) and impulsiveness (β = 0.56, [0.06, 1.05]). The associations did not change to any significant degree in the multivariate analysis (Model 2), and only the GS remained significantly associated with hyperactivity (β = 0.25, [0.12, 0.37]) and impulsiveness (β = 0.79, [0.20, 1.38]) when the gene-environment interaction term was added in the model (Model 3). Isolating the effect of earlier childhood experiences, we found that CM6 was associated with inattention (β = 0.46, [0.14, 0.78]), hyperactivity (β = 0.25, [0.05, 0.45]), and impulsiveness (β = 1.15, [0.25, 2.05]) in the univariate analysis. Only the association with inattention remained significant in the multivariate analysis, but it also disappeared when the gene-environment interaction term was added. No effects on the outcome variables were found for AEs and the gene-environment interaction terms. 

The gender-stratified analysis showed the gender difference in the results (Table 4). Among the females, CM and CM6 were associated with inattention, hyperactivity, and impulsiveness in the univariate and multivariate analyses. These associations were, however, not present among the males. Meanwhile, GS was only associated with hyperactivity across three different models.

## 6. Discussion

This study demonstrated that ACEs, particularly CM, may be associated with ADHD symptoms among a cohort of young people. Genetic factors, such as the MAOA, BDNF, and COMT genotypes, may also play a part in the associations among these outcomes. However, our analysis failed to demonstrate gene-environment interaction effects in this cohort.

Our results extended the association between childhood experiences and ADHD symptoms emerging later in young adulthood. Further dividing the ACE subtypes, we found childhood maltreatment, rather than adverse environments, to be significantly associated with ADHD symptoms. This finding stood in line with a prior Danish national study, which found a significant association between co-occurring multiple types of CM and higher levels of self-reported ADHD symptoms [10]. Another large Swedish twin registry found that CM was significantly associated with increased ADHD symptoms in adults, after accounting for familial confounding in the twin analysis [9]. Both studies also highlighted that certain CM types, particularly emotional, physical, and sexual abuses, were associated with adult ADHD symptoms [9,10]. Our data were unable to demonstrate the association between different CM types and adult ADHD symptoms, because nearly 40% of participants experienced multiple CM types at different points. Statistical significance was cancelled if multiple testing was corrected in the association analysis. Therefore, only cumulative types of ACEs were tested for their association with adult ADHD symptoms in the present study. One of the potential explanations for the association is that the effects of CM are likely to expose children to cumulative or chronic stress and in turn disturb molecular functions and neurodevelopment [33,34]. The non-significant association between AE and adult ADHD symptoms in our sample did not refute that AE was a stressor; instead, the way in which participants perceived these stressors may have differentially contributed to ADHD symptoms [35]. We also found that the association was only significant in females when the analysis was stratified by gender. Females tend to experience more heightened distress when being exposed to equal amounts of stressors [36]. This may explain our finding that females exposed to CM were likely to manifest ADHD symptoms in emerging adulthood. Again, it is beyond the scope of the present study, so that we did not examine the perception of stressors and how the perception of maltreatment may differ from the perception of adverse environments. Our argument may require further longitudinal research to explore the role of perceived stress after childhood adversity and how it impacts on neurodevelopment in the association with adult ADHD symptoms in both genders. However, a reverse causal inference may exist, as parents may have difficulties regulating their children’s behavior if their children manifest ADHD symptoms in early childhood. In order to prevent this putative mechanism, we separately analyzed the effect of CM6, and the finding was supportive of the hypothetical causal direction. Nonetheless, the item on the timeframe of CM did not allow us to differentiate between the adverse effects of early maltreatment and later abusive experiences if they were recurrent in late childhood and adolescence. This may in part explain why the estimates for the associations between CM and CM6 and outcome variables were very similar in the sensitivity analysis. Further research may consider separately looking at the effects of CM that occurs only in early childhood as compared to those only in late childhood or adolescence, so as to highlight the critical role of CM timeframe in developing adult ADHD symptoms. In our cohort, we found that only hyperactivity was significantly associated with the BDNF and COMT genes. This association was consistently found both in males and females. In line with previous research, differential associations with ADHD hyperactive and inattentive subtypes may reflect different neuropathophysiology in ADHD [18,37,38]. The non-significant association between selected SNPs and impulsiveness, a core ADHD symptom conventionally linked to hyperactivity, may also highlight that hyperactivity and impulsiveness are multifactorially inherited [3]. Although sharing some genetic factors, the genetic loading may be different in the trait hyperactivity-impulsiveness spectrum. Further, analysis using GS based on the cumulative number of risk alleles among the studied SNPs confirmed the genetic contribution of the hyperactivity subtype. However, negative findings for the association between GS and impulsiveness and inattention did not necessarily refute the genetic influences on these phenotypes. Since the subjects were generally healthy and recruited from the community, they were not able to represent those with more severe clinical cases. That might explain why the genetic association with impulsiveness and inattention subtypes found in prior research was not replicated in our study [18,39]. In addition, we assumed that the estimated effects for the genetic association with subclinical phenotypes were small in healthy subjects, and hence a larger number of subjects is required to improve the statistical power of the study. Further research may be needed to elucidate this assumption.

Contrary to our a priori assumption, we did not find any gene-environment interaction effect in association with ADHD symptoms. Since the interaction term was included in the regression model, only the main genetic effect remained significant in the model. Based on these results, we tentatively argued that these studied genes independently influence ADHD symptoms. Our results were supported by some other studies. For example, Retz et al. [40] did not find gene-environment interaction effects between psychosocial adversity and the COMT gene in an adult sample. Another study also pointed out that the interaction between the COMT gene and CM towards impulsiveness symptoms could be very trivial [41]. In a study examining the MAOA gene in a case-control group of maltreated children, Weder et al. found that aggressiveness may be moderated by the MAOA gene but only up to moderate levels of trauma exposure [42]. Inconsistent results may also be caused by a different set of pre-selected SNPs, despite the fact that they are harbored within the same gene. Given the mixed methodology and small effect sizes of gene-environment interaction studies, presumption-free and genome-wide data-driven studies may better address these issues and thereby provide more robust findings for gene-environment research [43].

The present study has some limitations. First, the items on the questionnaire were self-reported and thus subject to perception bias. For instance, our ADHD symptoms were screened merely using validated questionnaires that usually have a very limited space for a full list of the ADHD symptoms. Thus outcome variables may be biased if some other core symptoms were overlooked in a brief questionnaire. Participants may also not be aware of ADHD symptoms that actually occurred earlier in childhood, although we have excluded those who had prior diagnosis of ADHD. As such, professional evaluations, such as social workers’ reports on ACEs and physicians’ interviews on ADHD symptoms, may ameliorate this sort of bias. Obtaining additional information may affect the association estimates and this requires further research to confirm our findings. Worthy of attention is that our sample was made up of generally healthy young people. These findings may not be generalizable to those who meet full diagnostic criteria of ADHD. The causal interpretation should be cautious in a population with high levels of ADHD symptoms and childhood adversity. Second, only three SNPs were chosen to test the genetic and gene-environment interaction effects with ACEs on ADHD symptoms. Unselected SNPs may also play a role either due to their direct contribution or via their interaction with ACEs. Analysis of gene-wide SNPs identified by a thorough sequencing of these genes may further elucidate the genetic and gene-environment interaction effects of target genes on the outcome of interest. We only adjusted for age and gender, while some other contextual factors (e.g., socioeconomic status and parental education levels) that may be related to ADHD symptoms, were not adjusted. These factors have long been thought of as salient environmental factors of ADHD symptoms and thus they may play a part in the gene-environment interaction effect. However, the effects of these additional contextual factors may not easily be disentangled from those of ACEs given our limited sample size. Further research of a larger participation may be needed to explore the complex interplay of environmental factors.

## 7. Conclusions

CM was shown to have a significant impact on ADHD symptoms among a sample of young people, particularly in the case of females, while the combination genetic effects of MAOA (rs1137070 C > T), BDNF (rs6265 C > T), and COMT (rs4680 G > A), which reflected individual susceptibility, were significant in terms of the association with hyperactivity symptoms in both genders. Nonetheless, the gene-environment interaction was not observed in the present study. Our study highlights the roles of genetic and environmental factors in determining behavioral development in young people. The interplay between genes and environment, although non-significant in our analysis, can provide insight into predicting vulnerability to adult ADHD symptoms. Clinicians should be aware of the adverse impacts of ACEs on youth development because they may emerge as ADHD symptoms even over time. Genotypic information may help identify susceptible individuals who are at risk of ADHD symptoms; however, the clinical utility of genetic testing requires further research to validate such findings.

## Figures and Tables

**Table 1:** Demographic information of participants (N = 432).

Variable	Mean (SD) or N (%)
Age	20.0 (1.41)
Sex	
Male	221 (51.2)
Female	211 (48.8)
MAOA gene (rs1137070)	
CC	143 (33.1)
CT	104 (24.1)
TT Missing	178 (41.2) 7 (1.6)
BDNF gene (rs6265)	
CC	89 (20.6)
CT	195 (45.1)
TT Missing	107 (24.8) 41 (9.5)
COMT gene (rs4680)	
AA	31 (7.2)
AG	146 (33.8)
GG Missing	234 (54.2) 21 (4.9)
Outcomes	
Inattention (range: 4–16)	8.3 (2.4)
Hyperactivity (range: 2–8)	4.0 (1.5)
Impulsiveness (range: 15–60)	31.4 (6.7)
Childhood adversity	
AE (range: 0–8)	0.66 (0.98)
CM (range: 0–6)	0.50 (0.70)
CM6 (range: 0–6)	0.27 (0.53)

SD represents standard deviation; AE, adverse environment; CM, childhood maltreatment; CM6, childhood maltreatment before age 6 years.

**Table 2:** The association between MAOA, BDNF, and COMT polymorphisms and outcome variables.

Genotype or Allele	Inattention	Hyperactivity	Impulsiveness
MAOA gene (rs1137070)			
(CC + CT) vs TT	0.01 (−0.44, 0.47)	−0.24 (−0.52, 0.05)	−0.43 (−1.72, 0.87)
CC vs (CT + TT)	−0.01 (−0.49, 0.46)	−0.28 (−0.58, 0.01)	−1.36 (−2.70, −0.01) *
C vs T	−0.01 (−0.26, 0.25)	−0.16 (−0.32, 0.00)	−0.56 (−1.29, 0.18)
BDNF gene (rs6265)			
(CC + CT) vs TT	0.49 (−0.03, 1.02)	0.38 (0.06, 0.71) *	1.30 (−0.19, 2.80)
CC vs. (CT + TT)	0.07 (−0.49, 0.63)	0.35 (0.01, 0.70) *	1.22 (−0.36, 2.81)
C vs T	0.23 (−0.1, 0.56)	0.29 (0.08, 0.49) **	0.97 (0.04, 1.91) *
COMT gene (rs4680)			
(AA + GA) vs GG	−0.38 (−0.84,0.08)	−0.36 (−0.64, −0.07) *	−0.05(−1.37, 1.26)
AA vs (GA + GG)	−0.11 (−0.97,0.76)	0.14 (−0.40, 0.68)	0.11 (−2.35, 2.57)
A vs G	−0.26 (−0.60,0.08)	−0.24 (−0.45, −0.03) *	−0.09 (−1.06, 0.88)

*p* < 0.01 **; *p* < 0.05 *. Data are presented as β (95% confidence interval).

**Table 3:** Effects of genetic, environmental (childhood adversity) and gene-environment interaction on the outcome variables.

	Model 1 (Univariate)	Model 2	Model 3
Inattention			
AE	−0.10 (−0.33, 0.13)	−0.10 (−0.33, 0.12)	0.20 (−0.49, 0.90)
GS	0.14 (−0.04, 0.31)	0.14 (−0.04, 0.31)	0.19 (−0.02, 0.41)
AE × GS			−0.09 (−0.27, 0.10)
Hyperactivity			
AE	0.03 (−0.12,0.17)	0.02 (−0.12, 0.16)	0.20 (−0.23, 0.63)
GS	0.22 (0.11, 0.33) **	0.22 (0.11, 0.33) **	0.25 (0.12, 0.38) **
AE×GS			−0.05 (−0.16, 0.06)
Impulsiveness			
AE	0.40 (−0.25, 1.05)	0.39 (−0.25, 1.03)	1.53 (−0.43, 3.50)
GS	0.56 (0.06, 1.05) *	0.55 (0.06, 1.05) *	0.76 (0.16, 1.37) *
AE×GS			−0.32 (−0.83, 0.20)
Inattention			
CM	0.48 (0.16,0.79) **	0.48 (0.17, 0.80) **	0.77 (−0.16, 1.69)
GS	0.14 (−0.04,0.31)	0.12 (−0.04, 0.29)	0.17 (−0.04, 0.37)
CM × GS			−0.08 (−0.32, 0.16)
Hyperactivity			
CM	0.25 (0.06, 0.45) *	0.24 (0.05,0.43) *	0.48 (−0.09, 1.05)
GS	0.22 (0.11, 0.33) **	0.21 (0.11,0.32) **	0.25 (0.12, 0.37) **
CM × GS			−0.07 (−0.21, 0.08)
Impulsiveness			
CM	1.16 (0.26, 2.05) *	1.13 (0.23, 2.02) *	3.08 (0.45, 5.70)
GS	0.56 (0.06, 1.05) *	0.54 (0.04, 1.03) *	0.79 (0.20, 1.38) **
CM × GS			−0.54 (−1.22, 0.14)
Inattention			
CM6	0.46 (0.14, 0.78) *	0.46 (0.15, 0.78) *	0.71 (−0.22, 1.64)
GS	0.14 (−0.04, 0.31)	0.12 (−0.04, 0.29)	0.16 (−0.05, 0.37)
CM6 × GS			−0.07 (−0.31, 0.17)
Hyperactivity			
CM6	0.25 (0.05, 0.45) *	0.24 (0.04, 0.43)	0.44 (−0.13, 1.01)
GS	0.22 (0.11, 0.33) **	0.22 (0.11, 0.32) **	0.24 (0.11, 0.37) **
CM6 × GS			−0.06 (−0.20, 0.09)
Impulsiveness			
CM6	1.15 (0.25,2.05) *	1.12 (0.23,2.01)	2.83 (0.20, 5.46)
GS	0.56 (0.06,1.05) *	0.54 (0.04,1.03) *	0.76 (0.17, 1.34) *
CM6×GS			−0.47 (−1.15, 0.21)

*p* < 0.01 **; *p* < 0.05 *. Data are presented as β (95% confidence interval). GS represents genetic scores; AE, adverse environment; CM, childhood maltreatment; CM6, childhood maltreatment before age 6 years.

**Table 4:** Gender-stratified effects of genetic, environmental (childhood adversity) and gene-environment interaction on the outcome variables.

	Model 1 (Univariate)		Model 2		Model 3	
	Male	Female	Male	Female	Male	Female
Inattention						
AE	−0.18 (−0.51, 0.15)	0.00 (−0.31, 0.31)	−0.18 (−0.51, 0.15)	−0.01 (−0.32, 0.30)	0.29 (−0.72, 1.31)	0.03 (−0.93, 0.99)
GS	0.15 (−0.12, 0.42)	0.11 (−0.11, 0.32)	0.15 (−0.12, 0.42)	0.11 (−0.11, 0.32)	0.23 (−0.08, 0.53)	0.11 (−0.18, 0.41)
AE × GS					−0.13 (−0.39, 0.13)	−0.01 (−0.26, 0.24)
Hyperactivity						
AE	−0.01 (−0.21, 0.19)	0.07 (−0.13, 0.27)	−0.01 (−0.21, 0.18)	0.05 (−0.15, 0.25)	0.12 (−0.49, 0.73)	0.23 (−0.30, 0.76)
GS	0.26 (0.10, 0.42) **	0.18 (0.04, 0.31) *	0.26 (0.10, 0.42) **	0.18 (0.04, 0.31) *	0.28 (0.10, 0.47) **	0.21 (0.04, 0.39) *
AE × GS					−0.04 (−0.19, 0.12)	−0.05 (−0.19, 0.09)
Impulsiveness						
AE	0.45 (−0.53, 1.42)	0.35 (−0.48, 1.19)	0.46 (−0.51, 1.43)	0.33 (−0.50, 1.17)	1.30 (−1.70, 4.29)	1.85 (−0.71, 4.40)
GS	0.63 (−0.10, 1.36)	0.59 (−0.03, 1.20)	0.64 (−0.10, 1.37)	0.58 (−0.03, 1.2)	0.64 (−0.27, 1.55)	0.90 (0.10, 1.70) *
AE × GS					−0.23 (−1.01, 0.54)	−0.42 (−1.10, 0.25)
Inattention						
CM	0.26 (−0.20, 0.73)	0.68 (0.26, 1.10) **	0.25 (−0.22, 0.72)	0.69 (0.27, 1.11) **	0.69 (−0.74, 2.12)	0.89 (−0.22, 1.99)
GS	0.15 (−0.12, 0.42)	0.11 (−0.11, 0.32)	0.14 (−0.12, 0.41)	0.12 (−0.09, 0.33)	0.20 (−0.12, 0.52)	0.15 (−0.12, 0.43)
CM × GS					−0.11 (−0.47, 0.24)	−0.06 (−0.39, 0.26)
Hyperactivity						
CM	0.15 (−0.13, 0.44)	0.35 (0.08, 0.63) *	0.13 (−0.15, 0.41)	0.37 (0.10, 0.64) **	0.82 (−0.03, 1.67)	0.39 (−0.32, 1.11)
GS	0.26 (0.10, 0.42) **	0.18 (0.04, 0.31) *	0.26 (0.10, 0.42) **	0.19 (0.05, 0.32) **	0.35 (0.16, 0.54) **	0.19 (0.01, 0.37) *
CM×GS					−0.18 (−0.39, 0.03)	−0.01 (−0.22, 0.20)
Impulsiveness						
CM	0.61 (−0.78, 2.00)	1.72 (0.59, 2.85) **	0.56 (−0.82, 1.94)	1.72 (0.60, 2.84) **	2.87 (−1.35, 7.09)	2.81 (−0.45, 6.06)
GS	0.63 (−0.10, 1.36)	0.59 (−0.03, 1.20)	0.62 (−0.12, 1.35)	0.58 (−0.02, 1.19)	0.80 (−0.16, 1.75)	0.72 (0.01, 1.43) *
CM × GS					−0.60 (−1.65, 0.44)	−0.32 (−1.20, 0.57)
Inattention						
CM6	0.28 (−0.19, 0.74)	0.63 (0.21, 1.05) **	0.26 (−0.21, 0.73)	0.64 (0.22, 1.06) **	0.68 (−0.75, 2.11)	0.82 (−0.29, 1.93)
GS	0.15 (−0.12, 0.42)	0.11 (−0.11, 0.32)	0.14 (−0.12, 0.40)	0.12 (−0.09, 0.33)	0.20 (−0.12, 0.52)	0.15 (−0.12, 0.42)
CM6 × GS					−0.11 (−0.46, 0.25)	−0.06 (−0.38, 0.27)
Hyperactivity						
CM6	0.15 (−0.13, 0.44)	0.34 (0.07, 0.62) **	0.13 (−0.15, 0.41)	0.36 (0.09, 0.63) **	0.77 (−0.09, 1.62)	0.39 (−0.32, 1.10)
GS	0.26 (0.10, 0.42) **	0.18 (0.04, 0.31) *	0.26 (0.10, 0.42) **	0.18 (0.05, 0.32) *	0.34 (0.15, 0.54) **	0.19 (0.01, 0.37) *
CM6 × GS					−0.17 (−0.38, 0.04)	−0.01 (−0.22, 0.20)
Impulsiveness						
CM6	0.68 (−0.71, 2.06)	1.64 (0.51, 2.77) **	0.62 (−0.76, 2.00)	1.63 (0.51, 2.76) **	2.60 (−1.63, 6.83)	2.65 (−0.61, 5.90)
GS	0.63 (−0.10, 1.36)	0.59 (−0.03, 1.20)	0.61 (−0.13, 1.35)	0.58 (−0.02 1.19)	0.75 (−0.20, 1.70)	0.71 (0.00, 1.42) *
CM6					−0.51(−1.56, 0.53)	−0.29 (−1.18, 0.60)

*p* < 0.01 **; *p* < 0.05 *. Data are presented as β (95% confidence interval). GS represents genetic scores; AE, adverse environment; CM, childhood maltreatment; CM6, childhood maltreatment before age 6 years.
